# Lipid management in India: a nationwide, cross-sectional physician survey

**DOI:** 10.1186/s12944-017-0519-1

**Published:** 2017-07-03

**Authors:** Gurpreet S. Wander, Uday M. Jadhav, Amruta Chemburkar, Meena Lopez, Jaideep Gogtay

**Affiliations:** 1 Department of Cardiology, Hero DMC Heart Institute, Dayanand Medical College and Hospital, Ludhiana, Punjab India; 2 Department of Cardiology, MGM New Bombay Hospital, New Mumbai, 400703 India; 3 Cipla Ltd, Lower Parel, Mumbai, 400013 India

**Keywords:** Lipids, Survey, Physician survey, Dyslipidemia, India

## Abstract

**Background:**

Current international guidelines on dyslipidemia are not concordant on various aspects of management. Also, there are no uniformly accepted Indian guidelines. We, therefore, performed a physician survey to understand lipid management practices in India.

**Methods:**

An anonymous survey questionnaire was administered to gauge physicians’ self-reported behavior regarding lipid management aspects. Results were expressed in terms of percentages based on the number of responses obtained.

**Results:**

A total of 404 physicians participated in the survey. Eighty-eight percent respondents ordered a lipid profile before starting statin therapy, and 80% preferred to set lipid targets, though the tools used for calculating cardiovascular risk varied. Atorvastatin was preferred over rosuvastatin in primary prevention (72.9 vs. 32.4%), secondary prevention (54.6 vs. 46.7%), diabetic patients (56.3 vs. 40.3%) and post-ACS (78.3 vs. 34%). High-intensity statins were preferred by 73.7% of respondents in post-ACS cases. Fifty percent doctors chose not to use a statin in diabetic patients, irrespective of their LDL-C levels. The most preferred drug option for managing atherogenic dyslipidemia and moderate hypertriglyceridemia was statin-fibrate combination (55.1%) and fibrates (35.4%), respectively. Sixty-three percent doctors preferred to prescribe statins in patients with moderately high LDL-C and normal triglycerides, without CHD or CHD risk equivalents. Around 28% of doctors preferred not to use pharmacotherapy for managing isolated low HDL. Of the participants, 73% used fibrates in ≤20% of their dyslipidemic patients, with fenofibrate being the most preferred (90.5%). Ezetimibe was mainly used in patients with uncontrolled LDL-C despite statin therapy (52.4% respondents). Most preferred approaches to manage statin intolerance included reducing statin dose (39%) and stopping and restarting statins at a lower dose (34.5%). Fifty-two percent of doctors chose not to alter pre-existing therapy in patients who had LDL-C levels at goal but elevated non-HDL-C levels.

**Conclusion:**

This is the first survey in India that provides useful insights into Indian physicians’ self-reported perspectives on managing dyslipidemia in routine clinical practice. Despite concordance with the currently available guidelines in certain aspects, there is incongruence in managing specific dyslipidemia problems. Further continuing medical education and the development of evidence-based, India-specific lipid guidelines can help reduce some of these differences.

**Electronic supplementary material:**

The online version of this article (doi:10.1186/s12944-017-0519-1) contains supplementary material, which is available to authorized users.

## Background

Dyslipidemia or raised levels of blood lipids is one of the most important risk factors for development of cardiovascular disease (CVD) [1], a leading cause of mortality not only globally but also in India [2, 3]. Optimal management of dyslipidemia is, therefore, important to address the CVD burden. Guidelines on the management of dyslipidemia have been published by various international societies in the past. In 2013, the National Heart, Lung, and Blood Institute (NHLBI), U.S.A., in collaboration with the American College of Cardiology (ACC) and the American Heart Association (AHA), released guidelines that focused primarily on the risk of atherosclerotic cardiovascular disease (ASCVD) [4]. These guidelines abolished lipid targets recommended by the previous U.S. National Cholesterol Education Program (NCEP)–Adult Treatment Panel III (ATP III) guidelines [5], but identified four patient groups that would benefit from statin therapy. In 2014, the National Lipid Association (NLA) in the U.S. released recommendations that endorsed the use of lipid targets [6]. Besides disagreement on the utility of lipid targets, these guidelines are also not concordant on various other aspects of lipid management.

Though Indian guidelines for the management of dyslipidemia are not currently available, a consensus statement on the management of dyslipidemia in Indian subjects was published in 2014 [7]. In view of the varied recommendations from the different international associations and, especially the absence of India-specific guidelines, the approach to be followed by Indian clinicians to manage dyslipidemia patients in routine clinical practice remains unclear. Currently, there is lack of data on the lipid management practices followed by Indian clinicians in managing dyslipidemia in routine practice. We, therefore, conducted a survey to analyze physicians’ knowledge, attitudes and beliefs governing their decision-making in the management of lipids in clinical practice.

## Methods

An anonymous survey questionnaire (Additional file 1) was developed to gauge physicians’ self-reported behavior regarding different aspects of lipid management. The survey questionnaire consisted of 26 multiple-choice questions. The first four questions collected general information on participant demographics, education, and current practice setting. The next 11 questions were practice-related, and gathered information regarding lipid profile testing ordered by the physicians, their opinions pertaining to target low density lipoprotein cholesterol (LDL-C) goals, method used for CV risk stratification, and the statin preference in different practice settings (primary and secondary prevention, post-acute coronary syndrome [ACS] and diabetes). The last 11 questions of the survey were based on therapeutic approaches preferred by participants in patients of different dyslipidemic profiles, usage of non-statin drugs and their perspectives on statin intolerance and non-high-density lipoprotein cholesterol (HDL-C) goals. The study-related documents, including the survey questionnaire, were reviewed by two experienced cardiologists and approved by an ethics committee.

The survey questionnaire was administered to doctors attending continuing medical education (CME) programs in dyslipidemia on a single day in September 2015, at 23 centers across India. A duration of 30 min was allocated for completing the survey questionnaire during the CME program. Although most questions required a single response, multiple responses were allowed for specific questions.

Completed questionnaires from all the participants were compiled for analysis. A descriptive analysis of the collected data was performed by trained personnel and the results were expressed in percentages based on the number of responses (Additional file 2) obtained for each question. All respondents participated in the survey voluntarily, and no incentives were provided to the clinicians for their participation.

## Results

**Fig. 1:**
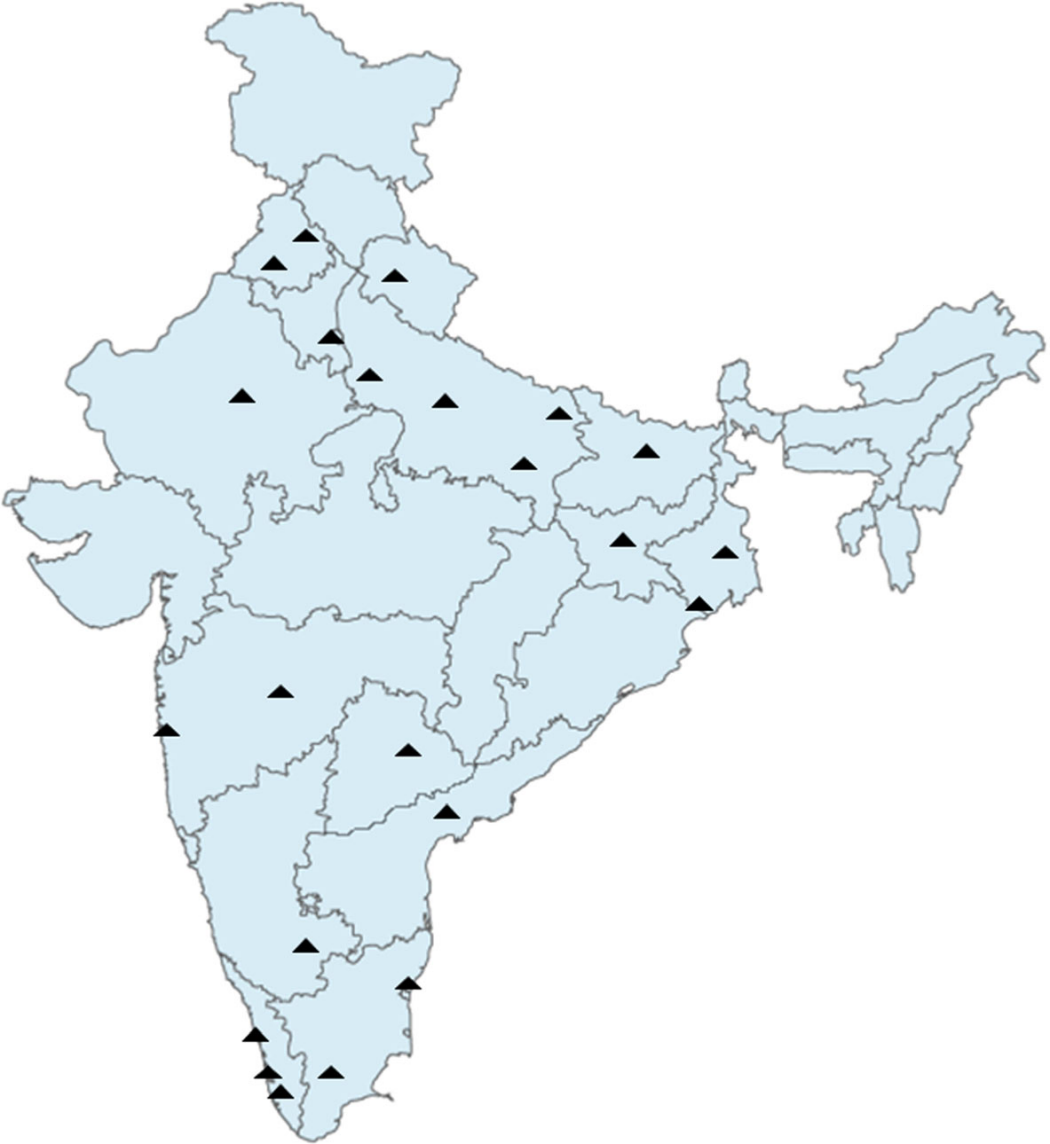
Survey sites across India

A total of 404 clinicians across 23 cities in India (Fig. 1) participated in the survey.

### Participant demographics

Fifty-five percent of the respondents had a qualification in internal medicine, while a quarter (26.1%) had a Bachelor of Medicine, Bachelor of Surgery (MBBS) degree. Among the rest, 5.5% were cardiologists and 7% had a super-specialization in diabetes, endocrinology or nephrology. Around 42% of the respondents were affiliated to academic institutions, 38% had a private practice, and 14% were practicing in corporate hospitals. A total of 309 (77.4%) respondents were under 50 years of age.

Around 65% of participants reported seeing up to 10 patients with dyslipidemia in their daily practice, while 21.7% reported treating 11–20 dyslipidemic patients on a daily basis.

### Lipid profile testing

**Fig. 2:**
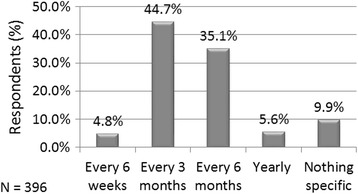
Frequency of lipid profile testing recommended by the study participants

Approximately 88% of doctors reported ordering a lipid profile test before initiating statin therapy. Eighty percent of the respondents recommended lipid profile testing to be done every 3 or 6 months in patients on lipid-lowering therapy (Fig. 2).

### Perspectives on LDL-C goals

**Fig. 3:**
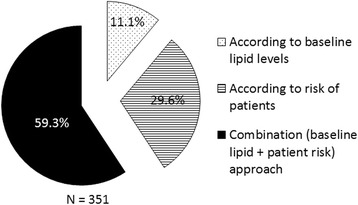
Approaches followed for setting LDL-C targets by the respondents

Around 80% of the respondents preferred to set LDL-C targets in their patients, with 59% preferring to use a combination of patient risk profile and baseline LDL-C to set an appropriate LDL-C target (Fig. 3).

Fifty-six percent of doctors opined that LDL-C targets should be lower in the Indian population than those recommended by the NCEP ATP III guidelines, while 15.6% of the respondents opted for retaining the same targets.

### Method for CV risk stratification

Different tools were preferred for stratifying CV risk in patients by the participants, with the most widely used (38.5% respondents) tool being the pooled cohort equation introduced in the ACC/AHA 2013 guidelines. A quarter of the respondents preferred the Framingham Risk Score endorsed by the NCEP ATP III guidelines while 21.1% favored the World Health Organization/International Society for Hypertension (WHO/ISH) risk prediction chart for South East Asian Indians, which is also recommended by the Indian consensus statement on dyslipidemia. Around 16% of the respondents chose to rely on their individual clinical impression rather than using any of the recommended risk stratification methods.

### Statin preference in specific clinical situations

**Fig. 4:**
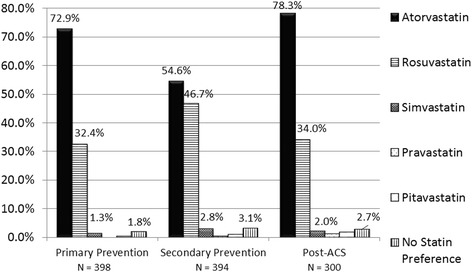
Choice of statin in different scenarios

The preference for atorvastatin for primary prevention, secondary prevention, and patients with ACS was the highest (72.9%, 54.6% and 78.3% of the respondents, respectively), followed by rosuvastatin (32.4%, 46.7% and 34%, respectively). In post-ACS cases, high-intensity statin therapy (atorvastatin 40/80 mg or rosuvastatin 20/40 mg) was preferred by 73.7% of the respondents. A substantially higher number of doctors preferred rosuvastatin for secondary prevention of CVD as compared to primary prevention (Fig. 4).

**Fig. 5:**
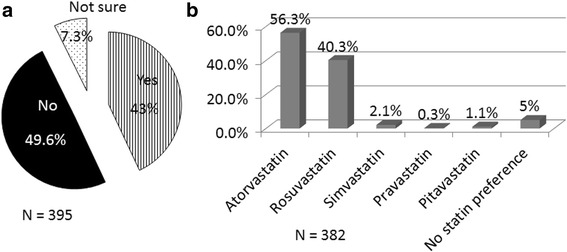
Statin use in diabetic patients. **a**: *Participant response on whether statins are preferred in all diabetics irrespective of their LDL-C levels* **b**: *Statin preference in diabetic patients*

Approximately half (49.6%) of the respondents chose not to use a statin in diabetic patients irrespective of their LDL-C levels (Fig. 5a). Atorvastatin was the statin of choice (56% of respondents) in diabetic patients, while 40% of doctors preferred rosuvastatin (Fig. 5b).

### Therapeutic approaches in specific dyslipidemia profiles

**Fig. 6:**
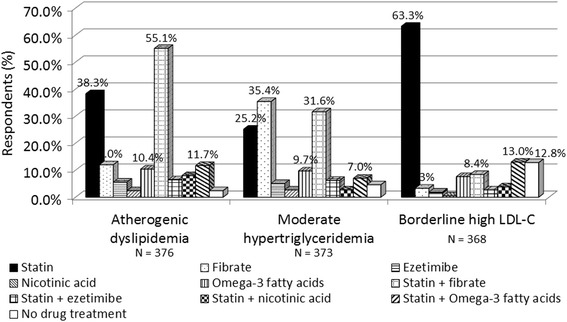
Choice of pharmacotherapy for different patient profiles. Atherogenic dyslipidemia: LDL-C > 160 mg/dL, TG 200–499 mg/dL and HDL-C < 40 mg/dL without CHD or CHD risk equivalents. Moderate hypertriglyceridemia: TG 200–499 mg/dL with LDL-C levels at goal. Borderline high LDL-C: LDL-C 130–160 mg/dL and TG (<200 mg/dL) without CHD or CHD risk equivalents

In patients with typical atherogenic dyslipidemia (LDL-C > 160 mg/dL, triglycerides [TG] 200–499 mg/dL and HDL-C < 40 mg/dL without coronary heart disease [CHD] or CHD risk equivalents), 55% of the respondents preferred a statin-fibrate combination while 38% preferred statin monotherapy (Fig. 6). For management of moderate hypertriglyceridemia (TG 200-499 mg/dL with LDL-C levels at goal), the approaches preferred were variable and included use of statins, fibrates and a statin-fibrate combination (Fig. 6). In patients with borderline high LDL-C levels (LDL-C 130–160 mg/dL) and normal TG (<200 mg/dL) without CHD or CHD risk equivalents, 63% of the respondents preferred to use statins (Fig. 6). Main approaches preferred for managing isolated low HDL-C (<40 mg/dL in males and <50 mg/dL in females) were statins (24.7%), omega-3 fatty acids (20.8%), nicotinic acid (20.5%) and fibrate or statin plus fibrate (10.5%); while 28.3% of respondents preferred no drug treatment.

*CHD* Coronary heart disease, *HDL-C* High density lipoprotein cholesterol, *LDL-C* low density lipoprotein cholesterol, *TG* triglycerides.

### Usage of non-statin drugs

Seventy-three percent of the participants preferred to use fibrates in ≤20% of their dyslipidemic patients while around 14% did not use fibrates in their clinical practice. Fenofibrate was the most preferred fibrate with 90.5% doctors preferring this drug, while 5.1% respondents preferred gemfibrozil and 2.9% preferred bezafibrate. Use of ezetimibe in patients with uncontrolled LDL-C despite moderate- or maximum-dose statin therapy was reported by 52.4% of the respondents, while 23.2% preferred it as an alternative drug in patients intolerant to statins (Table 1).

**Table 1:** Ezetimibe preference in different patient profiles

Patient profiles where ezetimibe is used	Number of respondents (%)
Patients with uncontrolled LDL-C despite maximum-dose statin therapy	107 (30.3)
Statin-intolerant patients	82 (23.2)
Patients with LDL-C not controlled by moderate-dose statin therapy	78 (22.1)
As monotherapy when LDL-C is slightly above goal	16 (4.5)
Patients with high total cholesterol and LDL-C	1 (0.3)
Do not use ezetimibe in clinical practice	119 (33.7)
TOTAL	353

*LDL-C* low-density lipoprotein cholesterol

### Perspectives on statin intolerance

About 92% of the respondents reported encountering statin intolerance in the range of 0–20% in their clinical practice. The most preferred option for management of statin intolerance was reducing statin dose (39% of the participants), closely followed by stopping and restarting statin at lower dose (34.5%) (Table 2).

**Table 2:** Preferred options for management of statin intolerance

Preferred strategy	Number of respondents (%)
Reducing statin dose	140 (39.0)
Stopping statin and restarting at a lower dose	124 (34.5)
Using alternative statin	80 (22.3)
Using non-statin drugs	39 (10.9)
Other (lifestyle modification)	5 (1.4)
TOTAL	359

### Perspectives on non-HDL-C targets

**Fig. 7:**
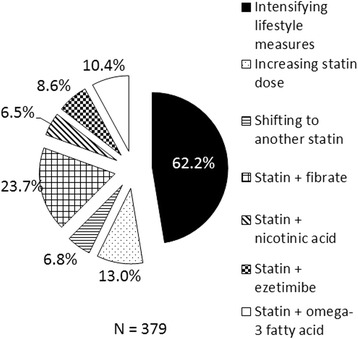
Approaches to attain non-HDL-C goals by clinicians. *HDL-C* High density lipoprotein cholesterol, *LDL-C* low density lipoprotein cholesterol

Fifty-two percent of the respondents preferred not to alter pre-existing therapy to attain non-HDL-C targets (non-HDL-C level of 30 mg/dL more than the LDL-C target [8]) in patients with LDL-C at goal. There was no clear consensus on the preferred strategy to attain non-HDL-C goals, with 62% of clinicians choosing intensification of lifestyle measures and around a quarter (23.7% respondents) opting to use statin-fibrate combination (Fig. 7).

## Discussion

This is the first survey that provides a unique insight into the various approaches followed by Indian clinicians for managing dyslipidemia in patients in routine clinical practice. We observed that there were some differences in the lipid management approaches currently followed by Indian clinicians.

Majority of the participants were likely to order lipid profile tests in patients prior to starting a statin prescription. The opinion on frequency of lipid profile testing (every 3–6 months) in patients already on lipid-lowering therapy was somewhat in line with the current recommendations. The ACC/AHA guidelines recommend a fasting lipid panel testing before initiating statin therapy and a repeat lipid panel testing at 4–12 weeks after statin initiation, after which assessments should be repeated every 3–12 months as clinically indicated [4]. The NLA Expert panel recommends lipoprotein lipid levels to be considered in conjunction with other ASCVD risk determinants for deciding treatment goals and strategies, and a re-evaluation of lipids in patients on statin therapy at every follow-up visit till the patient achieves the target goal after which response to therapy should be monitored periodically, within 4–12 months [8].

Majority of the participants chose to set LDL-C targets in their dyslipidemia patients. However, more than half of the doctors chose not to alter existing therapy in patients who had LDL-C levels at goal, but uncontrolled non-HDL-C levels. The latest ACC/AHA guidelines have abandoned the LDL-C and non-HDL-C goals recommended earlier by the NCEP ATP III guidelines due to lack of evidence from randomized controlled trials (RCTs) to support their continued use [4]. The NLA guidelines, on the other hand, continue to recommend LDL-C as well as non-HDL-C goals in patients with dyslipidemia, based on the argument that treatment goals are a useful means to ensure adequate aggressiveness of therapy and to maximize long-term adherence to treatment [6]. These guidelines proposed non-HDL-C as a better primary target for modification than LDL-C due to the stronger predictability of ASCVD morbidity and mortality of the former. The Indian consensus statement recommends LDL-C as the primary target for lipid-lowering therapy in patients with serum TG <500 mg/dL and suggests LDL-C target goals to be based on the available American and European guidelines owing to lack of prospective studies in the Indian population. It also endorses non-HDL-C as the primary treatment target when accurate estimation of LDL-C is not available and as a secondary target in patients with LDL-C at goal and TG > 200 mg/dL [7].

Among the respondents who set LDL-C targets in their patients, a majority used a combination approach based on baseline lipid levels along with ASCVD risk assessment—a practice in accordance with the recommendations of the NLA guidelines [8]. The Indian consensus statement too recommends setting target goals for patients as per the estimated global CV risk for deciding the appropriate management approach [7]. Most clinicians were of the opinion that lipid targets ought to be lower in Indian patients as compared to those recommended for Western counterparts by the NCEP ATP III guidelines. This indicates that Indian clinicians may perceive individuals of South Asian ethnicity to have a higher CV risk as compared to the Western population, as shown by a substantial number of studies, and, hence, recommend more aggressive targets [9]. However, currently, there are no prospective studies available to determine the optimal LDL-C goals and the treatment thresholds specifically in the Asian Indian ethnic population. Thus, the recommendations on optimum LDL-C goals and treatment thresholds continue to be based on the currently available Western guidelines [7].

Various approaches were used for CV risk stratification by the clinicians. Interestingly, a substantial number of respondents continued to use the Framingham Risk Score recommended by the NCEP ATP III guidelines, even though it has been replaced by the pooled cohort equation in the recently published ACC/AHA cholesterol-lowering guidelines. It is important to note that many ethnic groups, notably Asian Indians, have not been considered in this equation due to lack of RCT data involving these races. Many of the respondents also chose to rely on their individual clinical judgment for CV risk stratification in their patients. Plausible reasons for these different approaches may be the lack of a specific Asian Indian ethnicity option in the “race” section, due to which these patients would have to be included in the “Whites” or “Others” category in the pooled cohort risk calculator. Currently, there is no explicit CV risk stratification protocol recommended exclusively for the Indian or South Asian population. The Indian consensus statement also acknowledges that the currently available risk scoring algorithms have not been validated in the Indian population, nonetheless stating that the WHO/ISH risk prediction charts and the Joint British Society (JBS) risk prediction model may be more relevant to Indians [7].

The opinion amongst the respondents was divided on whether statins should be initiated in all diabetic patients, irrespective of their LDL-C levels. This may be due to lack of consensus between the various guidelines on this aspect. The ACC/AHA guidelines recommend statins for all diabetic patients without established ASCVD in the age group of 40 to 75 years with LDL-C levels >70 mg/dL. However, for diabetic patients who are <40 or >75 years of age, the guidelines recommend evaluation of the potential for ASCVD risk reduction and adverse effects, drug–drug interactions, and to give consideration to patient preferences prior to initiating, continuing, or intensifying statin therapy [4]. The guidelines from the American Diabetes Association (ADA) released in 2016 are in line with the ACC/AHA recommendations. These guidelines recommend statins for all diabetic patients between 40 and 75 years of age with no specific LDL-C cut-off levels. For patients >75 years of age, it recommends statin therapy to be individualized as per risk profile, whereas in patients <40 years of age, it recommends use of statins in established ASCVD [10]. The NLA recommendations, on the other hand, state that consideration should be given to use of statin therapy in patients with diabetes mellitus, irrespective of age as well as baseline atherogenic cholesterol levels [8].

Most of the respondents preferred potent statins (atorvastatin and rosuvastatin) for primary and secondary prevention of CVD in diabetics as well as in ACS patients, preferring the intensive dose of these statins post-ACS, which was in line with the available evidence and recommendations. There is a large body of evidence to support the use of atorvastatin [11, 12], as well as rosuvastatin [13] in different patient profiles. Additionally, only these two statins are included in the “high-intensity statins” category and also feature among the top two preferences in the list of “moderate-intensity statins”, in the ACC/AHA guidelines [4]. The NLA recommendations too include only these two statins in the “high intensity” category [6].

Clinicians reported use of different non-statin therapies to manage their dyslipidemic patients, with fibrates being the most common and fenofibrate being the most preferred fibrate. This may be due to the high prevalence of atherogenic dyslipidemia and hypertriglyceridemia in India [14, 15]. This approach is largely in line with the Indian consensus statement that recommends use of non-statin drugs, particularly fibrates, as an adjuvant to statins in case of persistent elevation of serum TG levels despite optimum lifestyle measures and statin therapy [7]. Fenofibrate, as monotherapy as well as in combination with statins, has been shown to significantly improve lipid profile (particularly TG and HDL-C levels) in patients with dyslipidemia versus placebo and gemfibrozil [16]. The international guidelines are not in agreement over the use of non-statin therapies. As per the ACC/AHA guidelines, non-statin drugs may only be considered in high-risk patients who show a less-than-anticipated response to statins, those who are unable to tolerate a less-than-recommended intensity of a statin, or who are completely statin-intolerant [4]. The ADA guidelines recommend ezetimibe for diabetic patients >40 years of age with ACS and LDL-C ≥ 50 mg/dL who cannot tolerate high-dose statins. It also states that statin and fenofibrate may be considered for men with both TG level ≥ 204 mg/dL and HDL-C level ≤ 34 mg/dL [10]. On the other hand, the NLA guidelines recommend non-statin therapies as an add-on to statins in patients with atherogenic dyslipidemia as well as in patients who have contraindications or are intolerant to statin therapy [6].

The different strategies chosen by the respondents for managing statin intolerance (reducing statin dose, stopping and restarting statin, using an alternative statin, and using non-statins) are in line with the ACC/AHA guidelines [4].

Similar physician-based surveys on lipid management practices in other countries have been published previously [17–19]. The results of these surveys revealed inconsistencies is the lipid management approaches as well as highlighted significant knowledge gaps in risk assessment and dyslipidemia management. Our study also found similar results in the Indian setting.

The present country-wide survey was conducted on the same day at 23 sites across India and obtained comprehensive insights from medical fraternities predominantly involved in managing dyslipidemia in clinical practice. Our study has a few limitations. The results were computed from the number of responses received for each question and there is a possibility that non-responding physicians have a knowledge base, practice patterns, and perceptions different from the respondents. However, the large sample size and the wide regional distribution of the survey may help mitigate some of these concerns. Also, this survey did not allow multiple responses to be selected for some questions, forcing the respondent to choose a “best fit” answer. Real-world practice may not be as precise. The cases experienced in clinical practice are unique and require an individualized management approach. Further, the response of the participants in our survey was not compared with recommendations outlined in the recently published European Society of Cardiology (ESC) guidelines [20], and an expert consensus statement from the Lipid Association of India on the management of dyslipidemia [21], as these publications were not available at the time of conduct of the survey.

Overall, we are of the opinion, that the treatment options preferred by the participants are largely reflective of the unique pattern of dyslipidemia observed in South Asian Indians (characterized by high levels of apolipoprotein [apo] B, TG and lipoprotein{a} [Lpa], borderline high levels of LDL-C; and low levels of HDL-C and apoA1) [22] and, hence, may not have completely conformed to the available international guidelines. Many of the management choices are in accordance with the recommendations suggested by the consensus statement on management of dyslipidemia in Indians [7]. Varied approaches are being adopted by physicians in certain aspects of dyslipidemia management, which may be due to their reliance on different guidelines that are non-representative of the Indian population, or on their individual clinical acumen.

## Conclusion

This is the first survey in India that provides useful insights into Indian physicians’ self-reported perspectives on managing dyslipidemia in routine clinical practice. The survey results reflect inconsistencies in the approach of Indian physicians, particularly with respect to CV risk estimation, utility of non-HDL targets, and usage of non-statin therapies. Conversely, there was relative unanimity in most aspects, namely, frequency of ordering lipid profile testing, setting of LDL-C target and usage of high-intensity statins. This survey accentuates the need for cohort studies for enabling the development of India-specific guidelines on dyslipidemia. It also emphasizes the importance of CME programs on dyslipidemia management with a view to ensuring a more consistent strategy for optimizing the management of dyslipidemia in India.

## Additional files

Additional file 1: Survey Questionnaire administered to the survey participants. (DOCX 25 kb)

Additional file 2: Number of respondents for each survey question. This data gives the number and percentage of participants responding to each survey question. (DOCX 14 kb)

## Abbreviations

ACC: American College of Cardiology
ACS: Acute Coronary Syndrome
ADA: American Diabetes Association
AHA: American Heart Association
Apo: Apolipoprotein
ASCVD: Atherosclerotic Cardiovascular Disease
ATP: Adult Treatment Panel
CHD: Coronary Heart Disease
CME: Continuing Medical Education
CVD: Cardiovascular Disease
ESC: European Society of Cardiology
HDL-C: High Density Lipoprotein Cholesterol
ICMR-INDIAB: Indian Council of Medical Research-India Diabetes
ISH: International Society of Hypertension
JBS: Joint British Society
LDL-C: Low Density Lipoprotein Cholesterol
Lp: Lipoprotein
MBBS: Bachelor of Medicine, Bachelor of Surgery
NCEP: National Cholesterol Education Program
NHLBI: National Heart, Lung, and Blood Institute
NLA: National Lipid Association
RCT: Randomized Controlled Trial
TG: Triglycerides
WHO: World Health Organization
