# Supplementary material for: Lipid management in India: a nationwide, cross-sectional physician survey
Source: Lipids Health Dis. 2017 Jul 3;16:130. doi: 10.1186/s12944-017-0519-1 (PMC5496264; doi:10.1186/s12944-017-0519-1)
Supplement: Supplementary file 1 — Survey Questionnaire administered to the survey participants. (DOCX 25 kb) [file 12944_2017_519_MOESM1_ESM.docx]

**Additional File 1**

**SURVEY QUESTIONNAIRE**

**City/Town: ___________________ State: _____________________**

**(Choose only one option unless specified. Please tick [√] appropriate option)**

1. **Qualification (choose the highest qualification)**
2. MBBS
3. MD (Internal Medicine)
4. DM _____________________ (Please specify specialization)
5. DNB _____________________ (Please specify specialization)
6. Other, please specify ______________________________
7. **Practice setting (please tick the predominant one)**
8. Private practice
9. Academic institution
10. Corporate hospital
11. Others, please specify__________________________________________
12. **Age group**
13. <30 years
14. 30–40 years
15. 41–50 years
16. 51–60 years
17. >60 years
18. **How many patients do you encounter with dyslipidemia in your daily practice?**
19. 0–5
20. 6–10
21. 11–20
22. 21–30
23. 31–40
24. >40
25. **How often do you ask your patients who are prescribed lipid-lowering therapy to undergo a lipid profile test?**
26. Every 6 weeks
27. Every 3 months
28. Every 6 months
29. Yearly
30. Nothing specific
31. **Do you order a lipid profile before prescribing statin therapy to patients with cardiovascular disease/risk factors?**
32. Yes, always
33. In most patients
34. In some patients
35. No
36. **Do you set any LDL-C target goals when you manage dyslipidemia in patients?**
37. Yes, always
38. In most patients
39. In some patients
40. No (if “No”, then directly go to Question 10)
41. **If yes, how do you decide LDL-C targets in your patients?**
42. According to baseline lipid levels
43. According to the risk of patients
44. Combination of “a” and “b” approach
45. **In your opinion, how should the LDL-C targets be in Indian patients as compared to those in the Western population?**
46. Same
47. Lower
48. Higher
49. Not sure
50. **Which method do you use to stratify cardiovascular risk in your patients?**
51. Framingham Risk Score
52. Atherosclerotic Cardiovascular Disease (ASCVD) risk calculator
53. World Health Organization (WHO) risk prediction chart for South East Asian Indians
54. Any other risk score, please specify ______________________________
55. Only my clinical impression is sufficient
56. **What is your choice of statin for primary prevention?**
57. Atorvastatin
58. Rosuvastatin
59. Simvastatin
60. Pravastatin
61. Pitavastatin
62. I do not have any particular statin preference
63. **What is your choice of statin for secondary prevention?**
64. Atorvastatin
65. Rosuvastatin
66. Simvastatin
67. Pravastatin
68. Pitavastatin
69. I do not have any particular statin preference
70. **Which is your preferred statin post-ACS? (Please specify dose used for the statin selected)**
71. Atorvastatin ______ mg
72. Rosuvastatin ______ mg
73. Simvastatin ______ mg
74. Pravastatin ______ mg
75. Pitavastatin ______ mg
76. I do not have any particular statin preference
77. **Do you prescribe a statin to all diabetic patients irrespective of their age and lipid profile?**
78. Yes
79. No
80. Not sure
81. **Which statin do you prefer for diabetic patients?**
82. Atorvastatin
83. Rosuvastatin
84. Simvastatin
85. Pravastatin
86. Pitavastatin
87. I do not have any particular statin preference
88. **What is your preferred drug option for patients with LDL >160 mg/dL, TG 200–499 mg/dL and HDL <40 mg/dL without CHD or CHD risk equivalents*? (Give order of preference in case of multiple options)**
89. Statin
90. Fibrate
91. Ezetimibe
92. Nicotinic acid
93. Omega-3 fatty acids
94. Statin + fibrate
95. Statin + ezetimibe
96. Statin + nicotinic acid
97. Statin + omega-3 fatty acids
98. None (no drug treatment)]
99. **What is your preferred drug option for patients with LDL levels at goal and TG 200–499 mg/dL? (Give order of preference in case of multiple options)**
100. Statin
101. Fibrate

*Diabetes mellitus, symptomatic carotid artery disease, peripheral artery disease, abdominal aortic aneurysm

1. Ezetimibe
2. Nicotinic acid
3. Omega-3 fatty acids
4. Statin + fibrate
5. Statin + ezetimibe
6. Statins + nicotinic acid
7. Statin + omega-3 fatty acids
8. None (no drug treatment)
9. **What is your preferred drug option for patients with LDL 130–160 mg/dL (borderline high) and normal TG (<200 mg/dL) without CHD or CHD risk equivalents*? (Give order of preference in case of multiple options)**
10. Statin
11. Fibrate
12. Nicotinic acid
13. Ezetimibe
14. Omega-3 fatty acids
15. Statin + fibrate
16. Statins + nicotinic acid
17. Statins + ezetimibe
18. Statin + omega-3 fatty acids
19. None (no drug treatment)
20. **What is your preferred drug option for patients with isolated low HDL (<40 mg/dL in males and <50 mg/dL in females)? (Give order of preference in case of multiple options)**
21. Statin
22. Fibrate
23. Nicotinic acid
24. Omega-3 fatty acids
25. Statin + fibrate
26. Statins + nicotinic acid
27. Statin + omega-3 fatty acids

*Diabetes mellitus, symptomatic carotid artery disease, peripheral artery disease, abdominal aortic aneurysm

1. None (no drug treatment)
2. **In what percentage of patients do you prescribe fibrate therapy?**
3. I do not use fibrates in my practice
4. 1–10%
5. 11–20%
6. 21–30%
7. 31–50%
8. 51–70%
9. >70%
10. **If yes, which drug from the fibrate class do you prefer in your practice?**
11. Fenofibrate
12. Gemfibrozil
13. Bezafibrate
14. I do not have any particular fibrate preference
15. **In which patients do you prescribe ezetimibe? (Multiple options can be chosen)**
16. Patients in whom LDL cannot be controlled by maximum-dose statin therapy
17. Patients in whom LDL is not controlled by moderate-dose statin therapy
18. As monotherapy in patients in whom LDL is slightly above goal
19. Patients intolerant to statins
20. I do not use ezetimibe in my practice
21. Any other cases, please specify
22. **What is the prevalence of statin intolerance^#^ seen in your practice?**
23. 0–10%
24. 11–20%
25. 21–30%
26. 31–40%
27. 41–50%
28. >50%

^#^ widely defined as not being able to tolerate a registered statin dose, due to side effects such as myalgia-myopathy, myositis or elevation of serum liver enzyme activities

1. **What is your preferred approach for patients presenting with statin intolerance^#^?**
2. Reduce statin dose
3. Stop statin therapy and restart later at a lower dose
4. Use alternative statin
5. Use non-statin drugs
6. Any other strategy, please specify
7. **Do you alter existing therapy to attain non-HDL-C goal in patients with LDL levels at goal?**
8. Yes, always
9. In most patients
10. In some patients
11. No
12. **If yes, then which is your preferred strategy to attain non-HDL-C goal in these patients?**
13. Intensifying lifestyle measures
14. Increasing the statin dose
15. Shifting to another statin
16. Statin + fibrate
17. Statin + nicotinic acid
18. Statin + ezetimibe
19. Statin + omega-3 fatty acids

^#^ widely defined as not being able to tolerate a registered statin dose, due to side effects such as myalgia-myopathy, myositis or elevation of serum liver enzyme activities
