# Supplementary material for: Lipid management in India: a nationwide, cross-sectional physician survey
Source: Lipids Health Dis. 2017 Jul 3;16:130. doi: 10.1186/s12944-017-0519-1 (PMC5496264; doi:10.1186/s12944-017-0519-1)
Supplement: Supplementary file 2 — Number of respondents for each survey question. This data gives the number and percentage of participants responding to each survey question. (DOCX 14 kb) [file 12944_2017_519_MOESM2_ESM.docx]

**Additional File 2**

**NUMBER OF RESPONDENTS FOR EACH SURVEY QUESTION**

| **Sr. No.** | **Survey Question** | **Number of Respondents** | **Percentage of Respondents (%)** |
| --- | --- | --- | --- |
| 1. | Qualification | 399 | 98.8 |
| 2. | Practice setting | 399 | 98.8 |
| 3. | Age group | 400 | 99 |
| 4. | Patients with dyslipidemia encountered in daily practice | 397 | 98.3 |
| 5. | Frequency of ordering a lipid profile test in patients on lipid-lowering therapy | 396 | 98 |
| 6. | Whether lipid profile test is ordered before starting statin therapy | 398 | 98.5 |
| 7. | Whether LDL-C target goals are set to manage dyslipidemia | 392 | 97 |
| 8. | If yes for the above question, how are targets decided? | 351 | 86.9 |
| 9. | Targets in Indian patients as compared to those in the Western population | 347 | 85.9 |
| 10. | Method used for stratifying CV risk in patients | 379 | 93.8 |
| 11. | Statin preference in primary prevention | 398 | 98.5 |
| 12. | Statin preference in secondary prevention | 394 | 97.5 |
| 13. | Statin preference in post-ACS patients | 300 | 74.3 |
| 14. | Whether statin is prescribed to diabetics irrespective of age and baseline lipid profile | 395 | 97.8 |
| 15. | Statin preference in diabetes | 382 | 94.6 |
| 16. | Pharmacotherapy preference in atherogenic dyslipidemia | 376 | 93.1 |
| 17. | Pharmacotherapy preference in hypertriglyceridemia | 373 | 92.3 |
| 18. | Pharmacotherapy preference in borderline dyslipidemia | 368 | 91.9 |
| 19. | Pharmacotherapy preference in isolated low HDL-C | 361 | 89.4 |
| 20. | Proportion of patients prescribed fibrates | 372 | 92.1 |
| 21. | Preference of fibrate | 397 | 98.3 |
| 22. | Patient profiles in which ezetimibe is prescribed | 353 | 87.4 |
| 23. | Prevalence of statin intolerance encountered in clinical practice | 363 | 89.9 |
| 24. | Preferred approach for managing statin intolerance | 359 | 88.9 |
| 25. | Whether existing therapy is altered to attain non-HDL-C goals | 360 | 89.1 |
| 25. | Preferred strategy to attain non-HDL-C goals | 379 | 93.8 |

ACS: acute coronary syndrome; CV: cardiovascular; LDL-C: low-density lipoprotein cholesterol; HDL-C: high-density lipoprotein cholesterol

Atherogenic dyslipidemia: LDL >160 mg/dL, TG 200–499 mg/dL and HDL <40 mg/dL without coronary heart disease (CHD) or CHD risk equivalents (Diabetes Mellitus, Symptomatic Carotid Artery Disease, Peripheral Artery Disease, Abdominal Aortic Aneurysm)

Borderline dyslipidemia: LDL 130–160 mg/dL and normal TG (<200 mg/dL) without CHD or CHD risk equivalents

Hypertriglyceridemia: TG 200–499 mg/dL with normal LDL-C

Isolated low HDL-C: <40 mg/dL in males and <50 mg/dL in females
